# A phase II study of FOLFIRINOX with primary prophylactic pegfilgrastim for chemotherapy-naïve Japanese patients with metastatic pancreatic cancer

**DOI:** 10.1007/s10147-021-02001-y

**Published:** 2021-08-08

**Authors:** Mitsuhito Sasaki, Hideki Ueno, Shuichi Mitsunaga, Akihiro Ohba, Hiroko Hosoi, Satoshi Kobayashi, Makoto Ueno, Tetsuji Terazawa, Masahiro Goto, Dai Inoue, Shin Namiki, Yasunari Sakamoto, Shunsuke Kondo, Chigusa Morizane, Masafumi Ikeda, Takuji Okusaka

**Affiliations:** 1 Department of Hepatobiliary and Pancreatic Oncology, National Cancer Center Hospital East, 6-5-1, Kashiwanoha, Kashiwa, Chiba 277-8577 Japan; 2 Department of Hepatobiliary and Pancreatic Oncology, National Cancer Center Hospital, Chuo-ku, Tokyo Japan; 3 Division of Hepatobiliary and Pancreatic Oncology, Kanagawa Cancer Center, Yokohama, Kanagawa Japan; 4 Cancer Chemotherapy Center, Osaka Medical College Hospital, Takatsuki, Osaka Japan; 5 Department of Gastroenterology and Hepatology, Tokyo Metropolitan Tama Medical Center, Fuchu, Tokyo Japan

**Keywords:** FOLFIRINOX, Febrile neutropenia, Pegfilgrastim, Pancreatic cancer

## Abstract

**Background:**

Although FOLFIRINOX is currently one of the standard therapies for chemotherapy-naïve patients with metastatic pancreatic cancer (MPC), the high rate of febrile neutropenia (FN) presents a clinical problem. This study aimed to evaluate the safety and efficacy of primary prophylactic pegfilgrastim with FOLFIRINOX in Japanese MPC patients.

**Methods:**

FOLFIRINOX (intravenous oxaliplatin 85 mg/m^2^, irinotecan 180 mg/m^2^, levofolinate 200 mg/m^2^, 5-fluorouracil (5-FU) bolus 400 mg/m^2^ and 5-FU 46 h infusion 2400 mg/m^2^) and pegfilgrastim 3.6 mg on day 4 or 5, every 2 weeks was administered to previously untreated MPC patients. The primary endpoint was the incidence of FN during the first 3 cycles. The planned sample size was 35 patients, but the trial was predefined to discontinue enrollment for safety if 4 patients developed FN.

**Results:**

At the enrollment of 22 patients, 4 patients developed FN in the first cycle, resulting in an incidence of FN of 18% {95% confidence interval [CI], 0.5–40.3%}, and enrollment was discontinued early. The incidence of grade 3 or higher neutropenia was 36.4%. Median relative dose intensities during the initial 3 cycles of oxaliplatin, irinotecan, bolus 5-FU, infusional 5-FU, and levofolinate maintained high (100%, 89.0%, 100%, 66.0%, and 100%, respectively). Response rate and median overall survival were 54.5% (95% CI 32.7–74.9) and 15.7 months (95% CI 7.9–18.8), respectively.

**Conclusions:**

This phase II study could not demonstrate any reduction in the incidence of FN, nevertheless some patients experience benefits for efficacy by maintaining dose intensity using prophylactic pegfilgrastim.

**Trial registration:**

http://www.umin.ac.jp/ctr/index-j.htm, UMIN000017538. Date of registration: May/13/2015

## Introduction

Pancreatic cancer (PC) is a highly lethal cancer with a 5-year survival rate of 2–3%, and is the fourth leading cause of cancer-related mortality in Japan [1]. As most patients are initially diagnosed with PC at an unresectable stage, chemotherapy plays the most important role in the treatment of advanced PC at presentation.

FOLFIRINOX [oxaliplatin, irinotecan, 5-fluorouracil (5-FU), and leucovorin] showed a significant improvement in overall survival (OS) compared to gemcitabine (GEM) for metastatic pancreatic cancer (MPC) in the ACCORD11/PRODIGE4 trial [2]. FOLFIRINOX has become the standard treatment for MPC. In Japan, a phase II trial (the LOHP-PII-05 trial) was conducted to evaluate the efficacy and safety of FOLFIRINOX in MPC patients [3]. This efficacy was similar to that reported in the ACCORD11/PRODIGE4 trial, but incidences of grade 3 or 4 toxicities, particularly neutropenia and febrile neutropenia (FN), were higher in the LOHP-PII-05 trial (77.8% and 22.2%, respectively) than in the ACCORD11/PRODIGE4 trial (45.5% and 5.4%, respectively). FN is a serious, potentially life-threatening toxicity and severe neutropenia and FN are reportedly observed more frequently in Asian populations, including Japanese, than in non-Asian populations [3–5]. Hence, there is a clinical need to reduce the incidence of FN in Japanese patients with MPC treated using FOLFIRINOX.

Granulocyte colony-stimulating growth factor (G-CSF) decreases the risk of FN in patients receiving myelosuppressive chemotherapies [6, 7]. According to several clinical oncology practice guidelines, prophylactic G-CSF is recommended when the risk of FN exceeds 20% [8–10]. Pegfilgrastim, a pegylated form of filgrastim, has a long half-life. A phase III placebo-controlled, double-blinded, randomized trial of pegfilgrastim in patients with breast cancer who received docetaxel in Europe and North America demonstrated significantly reduced incidences of FN, FN-related hospitalization, and antibiotic use [11]. Pegfilgrastim has, therefore, been approved in many countries. A recent systematic review of randomized clinical trials designed to investigate the impact of G-CSF on mortality and FN has revealed that prophylactic use of G-CSF reduces the rates of FN, dose reduction and treatment delay [12].

Prophylactic pegfilgrastim was expected to reduce the incidence of FN and allow maintenance of the dose intensity of FOLFIRINOX, and finally maximize the efficacy of FOLFIRINOX. We, therefore, conducted a phase II trial to evaluate the safety and efficacy of primary prophylactic pegfilgrastim in Japanese patients with MPC who received FOLFIRINOX.

## Patients and methods

### Study design

This open-label, single-arm phase II trial was conducted at 4 institutions. This clinical trial was conducted with the approval of the review boards of each participating institution and in accordance with the Declaration of Helsinki. This trial is registered with UMIN-CTR (http://www.umin.ac.jp/ctr/index-j.htm; identification number UMIN000017538).

### Patients

Inclusion criteria were as follows: histologically or cytologically confirmed pancreatic adenocarcinoma or adenosquamous carcinoma; Eastern Cooperative Oncology Group (ECOG) performance status (PS) 0 or 1; age 20–75 years; MPC with at least one measurable lesion; and adequate hematological, liver, and renal functions (hemoglobin ≥ 9.0 g/dL, white blood cell count ≤ 10,000/mm^3^, neutrophil count ≥ 2000/mm^3^, platelet count ≥ 100,000/mm^3^, total bilirubin ≤ upper limit of normal; aspartate transaminase and alanine transaminase ≤ 2.5 × upper limit of normal; and creatinine ≤ 1.2 mg/dL).

Patients were excluded if they had: received prior chemotherapy or radiation therapy; grade 2 or higher peripheral sensory neuropathy; blood transfusion, blood products, or hematopoietic growth factor preparations such as G-CSF within 7 days before enrollment; uridine diphosphate glucuronosyltransferase (UGT) genetic polymorphisms of homozygous UGT1A1*28 or UGT1A1*6 or heterozygous UGT1A1*6 and UGT1A1*28; apparent coelomic fluid (pleural effusion, ascites, or pericardial fluid) or peritoneal dissemination; diarrhea including watery stools within 3 days before enrollment; poorly controlled diabetes; synchronous or metachronous double cancer, excluding carcinoma in situ or intramucosal carcinoma cured by local treatment; active infection; or other serious concomitant diseases. All the above eligibility criteria were set in the same as the LOHP-PII-05 trial.

### Treatment

FOLFIRINOX was given every 2 weeks, as follows: oxaliplatin 85 mg/m^2^ infused over 120 min, immediately followed by levofolinate 200 mg/m^2^ infused over 120 min with the addition, after 30 min, of irinotecan 180 mg/m^2^ infused over 90 min, followed by 5-FU 400 mg/m^2^ bolus, followed by 2400 mg/m^2^ continuous infusion for 46 h. Subcutaneous injection of pegfilgrastim 3.6 mg was given on day 4 or day 5. Use of pegfilgrastim was mandated in the first 3 cycles, then each investigator chose whether to use pegfilgrastim in subsequent cycles.

A 5-HT3 receptor antagonist and dexamethasone were administered prior to FOLFIRINOX. Selective neurokinin 1 receptor antagonistic antiemetics were recommended to alleviate nausea and vomiting. Treatment was continued until disease progression, unacceptable toxicity, discontinuation as decided by the investigator, or patient refusal.

Chemotherapy was delayed until recovery from the following criteria: neutrophil count < 1500/mm^3^; platelet count < 75,000/mm^3^; total bilirubin > 1.5 mg/dL; grade 3 or higher peripheral sensory neuropathy; grade 2 or higher diarrhea; and watery stools. When a predefined toxic event in the protocol occurred, dose adjustment was required. The reduced dose was set at 150 mg/m^2^ and 120 mg/m^2^ for irinotecan, 65 mg/m^2^ and 50 mg/m^2^ for oxaliplatin, and 1800 mg/m^2^ and 1200 mg/m^2^ for infusional 5-FU.

### Outcomes and assessments

The primary endpoint was the incidence of FN during the first 3 cycles of chemotherapy. FN was defined in this study as a single body temperature measurement > 38.3 °C, or a temperature ≥ 38.0 °C sustained over 1 h, with a neutrophil count < 1000/mm^3^ within 1 day of the identified fever. According to the LOHP-PII-05 study [3], all patients who developed FN developed the adverse reaction during the first cycle of therapy, and more than half of the patients required dose reduction of either oxaliplatin or irinotecan due to the development of adverse events during the first 3 cycles of treatment. Hence, the evaluation period was set to be during the first 3 cycles of therapy.

Secondary endpoints were OS, progression-free survival (PFS), response rate (RR), and relative dose intensity (RDI) during the first 3 cycles of chemotherapy, and safety for all patients. OS was defined as the time from study enrollment to death from any cause. PFS was defined as the time from study enrollment to disease progression or death. RDI was defined as the proportion of delivered dose intensity to the planned dose intensity for each agent from first day of first cycle to last day of third cycle. Patients were evaluated for toxicities during the entire course. Complete blood counts, blood chemical tests, and physical examinations were carried out at least every 2 weeks. Computed tomography was performed at least every 6 weeks. RR was assessed based on Response Evaluation Criteria in Solid Tumors (RECIST) version 1.1 guidelines. Toxicities were graded according to Common Terminology Criteria for Adverse Events (CTCAE) version 4.0.

### Statistical analysis

Incidence of FN during the first 3 cycles of pegfilgrastim with FOLFIRINOX was expected to be lower than that from the LOHP-PII-05 trial (22.2%). A minimum sample size of 32 was required for a one‐sided α of 0.1 and *β* of 0.2, with an expected FN incidence of 5% and a threshold incidence of 20% using the binomial test. The target sample size was set at 35 patients, considering that ineligible patients would be enrolled in this trial. If more than 4 of the 35 patients were to develop FN, the upper limit of the 95% confidence interval (CI) for FN incidence would be > 20%. As a result, enrollment was to be stopped immediately for the safety of patients as soon as FN was identified in 4 patients. The Kaplan–Meier method was used to estimate survival curves. All statistical analyses were performed with EZR ver.1.51 (Saitama Medical Center, Jichi Medical University, Saitama, Japan), which is a graphical user interface for R (The R Foundation for Statistical Computing, Vienna, Austria).

## Results

### Patient characteristics

Between May 2015 and July 2016, a total of 22 patients were enrolled from the 4 institutions. FN was identified in a fourth patient after enrollment of 22 patients, and further enrollment was, therefore, suspended.

Patient characteristics at baseline are shown in Table 1. Median age was 61 years (45–73 years), and 63.6% of patients had PS 0. The primary site of the tumor was the head of the pancreas in 68.2% of patients and the most common site of metastasis was the liver. A biliary stent was present in 22.3% of patients and 54.5% of patients showed a heterozygous *UGT1A1* genotype.

**Table 1:** Baseline characteristics

Characteristics	*n* (%)
Sex	
Male	12 (54.5)
Female	10 (45.5)
Age, years	
Median	61
Range	45–73
< 65	15 (68.2)
≥ 65	7 (31.8)
ECOG PS	
0	14 (63.6)
1	8 (36.4)
Body surface area (m^2^)	
Median	1.63
Range	1.22–1.92
Primary tumor location	
Head	15 (68.2)
Body and/or tail	7 (31.8)
Metastatic site	
Liver	13 (59.0)
Lymph nodes	4 (18.2)
Lung	4 (18.2)
Peritoneum	3 (13.6)
Biliary stent placement	6 (27.3)
UGT1A1 (*6/*28)	
Wild/wild	10 (45.5)
Wild/heterozygous, heterozygous/wild	12 (54.5)
CA19-9 (ng/ml)	
Median	1703
Range	0–33,060

*ECOG PS* Eastern Cooperative Oncology Group performance status, *UGT1A1* uridine diphosphate glucuronosyltransferase 1A1, *CA19-9* carbohydrate antigen 19-9

### Treatment exposure

All patients received the study drugs, and 20 patients completed 3 cycles of treatment. The median number of treatment cycles was 9 (1–41). Median relative dose intensities during the first 3 cycles of oxaliplatin, irinotecan, bolus 5-FU, infusional 5-FU, and levofolinate were 100%, 89.0%, 66.0%, 100%, and 100%, respectively. Dose reductions and/or treatment delays occurred in 14 patients (63.6%) during the first 3 cycles. The most common reasons for treatment delay and/or dose reductions were FN (4 patients), diarrhea (3 patients), and investigator decision (serum C-reactive protein elevation, 2 patients; serum aminotransferase elevation, 2 patients). The major reasons for treatment discontinuation were disease progression (14 patients, 63.6%) and adverse events (5 patients, 22.7%: biliary tract infection, 2 patients; FN, 1 patient; catheter-related infection, 1 patient; diarrhea, 1 patient).

### Safety

Four patients developed FN during the first 3 cycles (18.2%; 95% CI 0.5–40.3%). Detailed characteristics of these four patients are shown in Table 2. All instances of FN developed during the first cycle, but no other specific clinical features were observed in these patients.

**Table 2:** Characteristics of patients who developed febrile neutropenia

Characteristics	1	2	3	4
Sex	Male	Male	Female	Female
Age (years)	60	66	65	72
ECOG PS	1	0	0	1
Primary tumor location	Head	Head	Body	Head
Biliary stent	+	−	−	+
UGT1A1 polymorphism	Wild type	Wild type	*6 Hetero	Wild type
White blood cells (/mm^3^)	5030	7600	4300	3700
Neutrophils (/mm^3^)	3093	5342	2570	2880
Lymphocytes (/mm^3^)	1006	1452	1380	560
Platelets (× 10^4^/mm^3^)	23	21.3	16.5	25.6
Albumin (g/dL)	3.6	3.9	4.1	3.4
Creatinine (mg/dL)	0.71	0.73	0.54	0.49
Febrile neutropenia, onset	Cycle 1, day 9	Cycle 1, day 8	Cycle 1, day 8	Cycle 1, day 9
Neutrophil, nadir (/mm^3^)	218	50	20	130
Neutropenia grade 3–4, duration (days)	4	2	5	5
Best response	Stable disease	Stable disease	Partial response	Partial response
Progression-free survival (months)	9.6	2.6	13.3	5.9
Survival (months)	19.1	8.5	21.9	7.0

*ECOG PS* Eastern Cooperative Oncology Group performance status, *UGT1A1* uridine diphosphate glucuronosyltransferase 1A1

Treatment-related adverse reactions occurring in all 22 patients are summarized in Table 3. Treatment-related serious adverse events occurred in 5 patients (22.7%), comprising FN in 4 patients and grade 3 diarrhea in 1 patient. Grade 3–4 toxicities occurred in 12 patients (54.5%). The major grade 3–4 hematological toxicities were neutropenia (36.4%), leucopenia (36.4%), and thrombocytopenia (22.7%), and the major grade 3–4 non-hematological toxicities were anorexia (13.6%), diarrhea (4.5%), nausea (4.5%), and peripheral neuropathy (13.6%). Bone pain is the common adverse event of interest for pegfilgrastim, but occurred in only 1 patient (4.5%) in this study. No treatment-related deaths were encountered.

**Table 3:** Adverse events

	Present trial		LOHP-PII-05 trial [3]	
	Any grade, *n* (%)	Grade 3/4, *n* (%)	Any grade, *n* (%)	Grade 3/4, *n* (%)
Hematological toxicities				
Neutropenia	9 (40.9)	8 (36.4)	34 (94.4)	28 (77.8)
Leukopenia	9 (40.9)	8 (36.4)	33 (91.7)	16 (44.4)
Anemia	21 (95.4)	1 (4.5)	31 (86.1)	4 (11.1)
Thrombocytopenia	20 (90.9)	5 (22.7)	32 (88.9)	4 (11.1)
Non-hematological toxicities				
Febrile neutropenia	4 (18.4)	4 (18.4)	8 (22.2)	8 (22.2)
Nausea	17 (77.3)	1 (4.5)	32 (88.9)	3 (8.3)
Anorexia	16 (72.7)	3 (13.6)	31 (86.1)	4 (11.1)
Diarrhea	15 (68.2)	1 (4.5)	31 (86.1)	3 (8.3)
Fatigue	13 (59.0)	0 (0)	16 (44.4)	0 (0)
Peripheral sensory neuropathy	15 (68.2)	3 (13.6)	27 (75)	2 (5.6)
Fever	7 (31.8)	0 (0)	9 (25)	0 (0)
Bone pain	1 (4.5)	0 (0)	NA	NA
Elevated ALP	19 (86.4)	0 (0)	15 (41.7)	3 (8.3)
Elevated AST	17 (77.3)	0 (0)	20 (55.6)	2 (5.6)
Elevated ALT	17 (77.3)	0 (0)	20 (55.6)	3 (8.3)

*ALP* alkaline phosphatase, *AST* aspartate aminotransferase, *ALT* alanine aminotransferase, *NA* not available

### Efficacy

Response was classified as partial response in 12 patients, stable disease in 8 patients, and progressive disease in 2 patients. The overall RR and disease control rate in all patients were 54.5% (95% CI 32.7–74.9%) and 90.1% (95% CI 69.4–98.4%), respectively. One patient achieved complete response of peritoneal metastases under this regimen, and underwent distal pancreatectomy and splenectomy for the primary tumor. In this case, pathological findings revealed R0 and he remained alive without recurrence for 2 years.

**Fig. 1:**
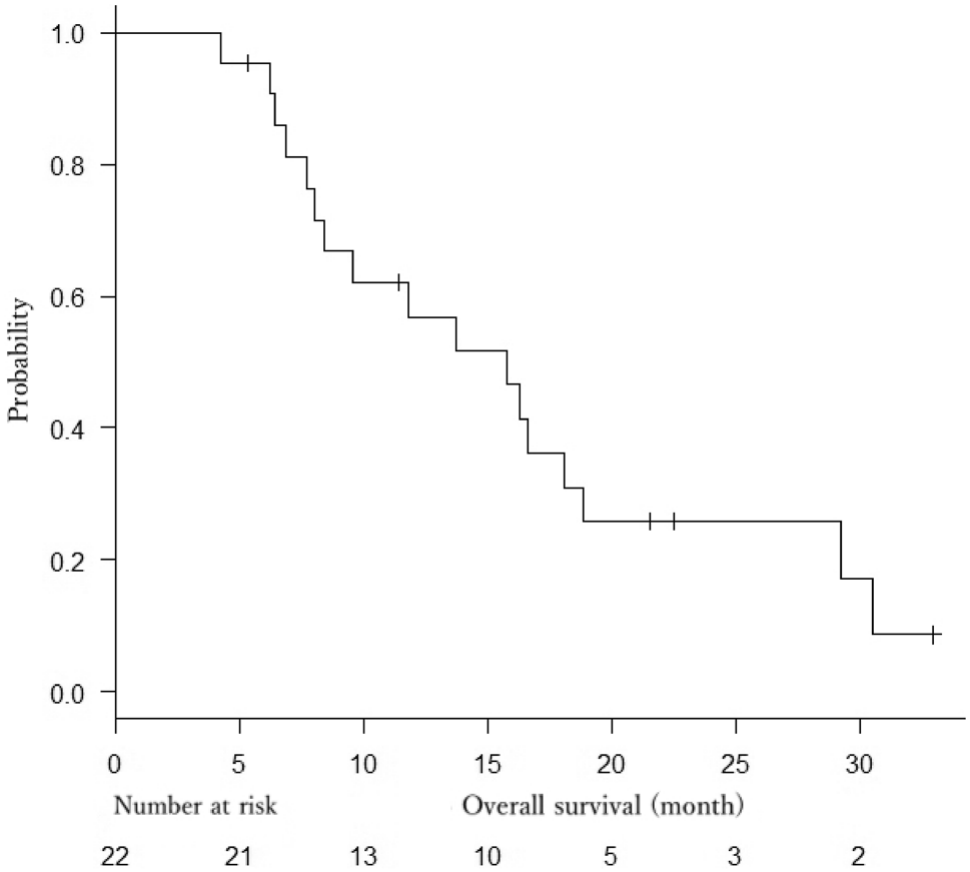
Kaplan–Meier estimate of overall survival

**Fig. 2:**
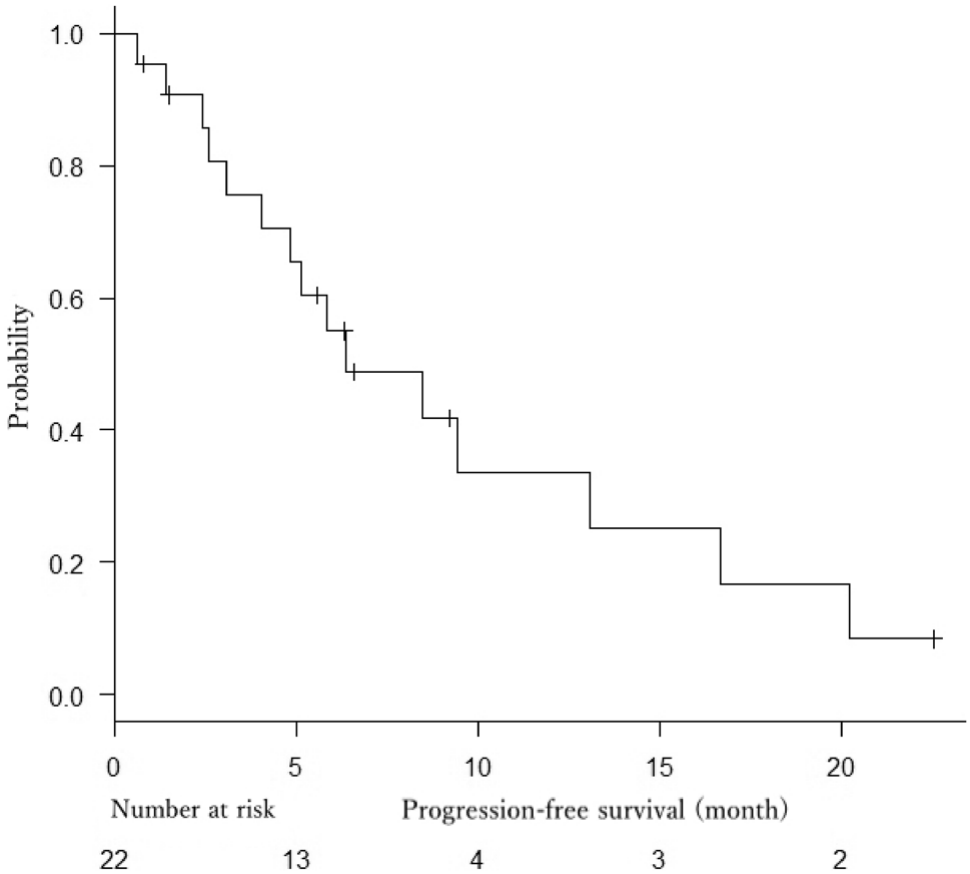
Kaplan–Meier estimate of progression-free survival

At the time of analysis, 19 patients had died, 3 patients were alive, and no patients had been lost to follow-up. Median OS was 15.7 months (95% CI 7.9–18.8 months) (Fig. 1), and median PFS was 6.4 months (95% CI 4.0–13.0 months) (Fig. 2).

Of the 22 enrolled patients, 19 received second-line therapy. The most common main treatments were GEM + nab-paclitaxel (11 patients) and GEM alone (2 patients).

## Discussion

To the best of our knowledge, this represents the first prospective study to evaluate the safety and efficacy of primary prophylactic pegfilgrastim in Japanese MPC patients who received FOLFIRINOX. Current clinical practice guidelines [8–10] recommend primary prophylactic G-CSF when the risk of FN is > 20% in chemotherapy. An incidence of FN > 20% was reported in the LOHP-PII-05 trial [3], so the present study was conducted in expectation of a reduced incidence of FN with FOLFIRINOX for Japanese MPC patients. However, FN developed in 4 of the 22 patients (18.0%) during the first 3 cycles regardless of primary prophylaxis with pegfilgrastim. Our study thus failed to demonstrate a reduction in the incidence of FN by adding pegfilgrastim to FOLFIRINOX.

Patient characteristics also represent important risk factors for FN. Various retrospective studies have reported female sex, body mass index ≥ 25 kg/m^2^, biliary stent insertion, low pretreatment platelet count and *UGT1A1* genetic polymorphisms as risk factors for FN in Asian PC patients treated with FOLFIRINOX [13, 14]. In addition, a proportion of patients with other malignancies still develop FN despite receiving prophylactic G-CSF. Lee et al. reported low pretreatment platelet count (< 15 × 10^4^/μL) as a risk factor for FN among cancer patients with pegfilgrastim prophylaxis [15]. However, the 4 patients who developed FN in this study showed none of these features. We thus could not identify any specific cause for the failure to reduce the incidence of FN in the features of these patients.

Combination use of prophylactic pegfilgrastim with chemotherapy could contribute to reducing the frequency of grade 3–4 neutropenia and maintaining RDI [10]. Neutropenia was the most frequent cause for reducing RDIs of FOLFIRINOX in the LOHP-PII-05 trial. The frequency of grade 3 or 4 neutropenia in this study (36.4%) was lower than that in the LOHP-PII-05 trial (77.8%). Thus, the major reason for reducing RDIs was not neutropenia but was non-hematological toxicity in this study. In fact, RDIs during 3 cycles in this study (oxaliplatin 100%, irinotecan 89.0%, bolus 5-FU 66.0%, infusional 5-FU 100%, l-leucovorin 100%) were maintained high.

With regard to non-hematological toxicities, pegfilgrastim appeared well tolerated. The common adverse events associated with pegfilgrastim were bone pain (4.5%) and ALP elevation (86.4%), and no patients showed grade 3 or higher adverse events related to pegfilgrastim. The incidences of grade 3 or higher anorexia, nausea, diarrhea, and peripheral sensory neuropathy in this study resembled those in the LOHP-PII-05 trial (13.6%, 4.5%, 4.5% and 13.6% vs 11.1%, 8.3%, 8.3% and 5.6%, respectively). These results suggest that pegfilgrastim use did not increase the risk of non-hematological toxicities.

In our study, RR, DCR, PFS, and OS were 54.5%, 90.1%, 6.4 months, and 15.7 months, respectively. These results were better than those of both the ACCORD11/PRODIGE4 trial (31.6%, 70.2%, 6.4 months and 11.1 months, respectively) and the LOHP-PII-05 trial (38.9%, 69.4%, 5.6 months, and 10.7 months, respectively). In some types of cancer such as lung, breast, and ovarian cancers, maintaining RDI has been shown to improve clinical outcomes [16–19]. Regarding FOLFIRINOX for advanced PC, a few retrospective studies have reported the effect of RDI on efficacy. Lee et al. reported that a cumulative RDI of FOLFIRINOX > 70% was related to radiological response [20] and Kobayashi et al. reported that a high RDI (> 75%) for irinotecan within the first 2 cycles correlated positively with objective response [21]. The favorable RR in our study may have been obtained by maintaining RDI with primary prophylactic pegfilgrastim. On the other hand, while the most common second-line treatment in the ACCORD11/PRODIGE4 and LOHP-PII-05 trials was GEM alone, in our study, many patients received gem + nab-paclitaxel as second-line treatment. Thus, the favorable survival may have been influenced by the content of the second-line treatments.

Recently, some prospective and retrospective studies were conducted to evaluate the efficacy and safety profile of modified FOLFIRINOX therapy in patients with advanced PC. Ozaka et al. reported that the modified FOLFIRINOX regimen, in which bolus 5-FU is omitted and the dose of irinotecan is reduced (to 150 mg), without prophylactic pegfilgrastim administration, shows an improved safety profile with maintained efficacy in Japanese patients with MPC [22]. Based on this report, this modified FOLFIRINOX regimen has been used as the domestic standard in Japan. The modified FOLFIRINOX regimen including prophylactic pegfilgrastim administration has been reported from western countries to yield favorable results [23, 24]. With respect to adverse events other than FN, the incidence of grade 3 or higher thrombocytopenia in our study was higher than that in the Japanese modified FOLFIRINOX study (22.7 vs. 2.9%). Kajiyama et al. reported from a large national database, that pegfilgrastim increased the risk of thrombocytopenia in Japanese patients treated with antineoplastic agents [25]. Therefore, as not only the doses of FOLFIRINOX, but also the dose of pegfilgrastim could have had an influence on the incidence of severe thrombocytopenia, careful attention should be paid when adding pegfilgrastim administration to the modified FOLFIRINOX regimen for Japanese patients with PC.

The major limitation of the present study was that we used pegfilgrastim at a dose of 3.6 mg. According to a trial that verified the dose response of pegfilgrastim in Japanese patients, efficacy peaked at 3.6 mg [26]. The fixed dose of prophylactic pegfilgrastim in Japanese patients is 3.6 mg, as the dose approved by the Japanese healthcare system. On the other hand, pegfilgrastim is usually used at a dose of 6.0 mg in other countries, and Faris et al. reported that prophylactic pegfilgrastim at 6.0 mg with FOLFIRINOX for PC reduced the incidence of FN [27]. Hence, while pegfilgrastim 3.6 mg failed to reduce FN with FOLFIRINOX in Japanese MPC according to the present results, whether pegfilgrastim 6.0 mg could reduce FN remains unclear.

In conclusion, this phase II study was unable to demonstrate a reduction in the incidence of FN using primary prophylactic pegfilgrastim with FOLFIRINOX in Japanese MPC. However, some patients benefited from maintaining chemotherapy dose intensity by prophylactic pegfilgrastim. The combination of prophylactic pegfilgrastim may contribute to reducing the rate of neutropenia and maintaining RDI, and could lead to favorable antitumor effects. Further studies are warranted to determine the optimal use of primary prophylactic pegfilgrastim with FOLFIRINOX for Japanese PC patients.
